# Therapeutic use of cold-stored platelets in humans: protocol for a systematic review and meta-analysis

**DOI:** 10.1136/bmjopen-2025-113548

**Published:** 2026-06-29

**Authors:** Chloë E George, Thomas Scorer, Anne Cleves, Ioannis Pilavakis, Shaun Harris, Kym Carter

**Affiliations:** 1 Welsh Blood Service, Pontyclun, UK; 2 Centre of Defence Pathology, Royal Centre for Defence Medicine, Birmingham, UK; 3 Velindre University NHS Trust Library, Velindre Cancer Centre, Cardiff University, Cardiff, UK; 4 Ioannis Pilavakis, Emergency Medical Retrieval & Transfer Service, Cymru, UK; 5 Swansea Trials Unit, Swansea University Medical School, Swansea, UK

**Keywords:** Systematic Review, Blood bank & transfusion medicine, HAEMATOLOGY

## Abstract

**Abstract:**

**Introduction:**

Interest in cold-stored platelets (CSP) has seen a resurgence over the last 20 years, having not been clinically used since the 1980s. A multitude of in vitro studies have reported positive characteristics such as improved haemostatic function and longer storage duration in CSP compared with their room-temperature-stored counterparts. More recently, CSP have been investigated in clinical trials and used to improve supply chain resilience in some settings. This systematic review and meta-analysis will synthesise and describe the published evidence on the therapeutic use of CSP to determine the feasibility, safety and efficacy of their use and identify key areas for future research.

**Methods and analysis:**

The Medline, Cochrane and EMBASE databases will be searched from inception to date of search for all studies including therapeutic transfusions of CSP in the English language as well as grey literature searches. Abstract screening and shortlisted full-text screening will be performed by two independent reviewers, adjudicated by a third reviewer. The data will be extracted by two reviewers and a narrative review formulated. Risk of bias will be assessed using the risk-of-bias tool for randomised trials (RoB-2) and the Risk of Bias In Non-randomized Studies - of Interventions tool. A meta-analysis will be undertaken using fixed-effect or random-effects models, contingent on the degree of statistical heterogeneity, as quantified by the I² statistic.

**Ethics and dissemination:**

No ethical review is required as no original data will be collected. Results will be disseminated through peer-reviewed publication and at academic conferences.

**Systematic review registration:**

PROSPERO CRD420251086610.

**STRENGTHS AND LIMITATIONS**

- The protocol follows the Cochrane systematic review protocol methodology.
- This review will include randomised and non-randomised controlled studies to capture all relevant evidence regarding therapeutic use of cold-stored platelets (CSP) and its safety profile.
- We plan to meta-analyse the outcome data; however, if heterogeneity is too substantial across the included studies, a narrative synthesis may need to be undertaken.
- A limitation of the review is that it will only include literature published in the English language.
- The review will not include any subgroup comparison of CSP-related data across different storage temperature ranges.

## Background

 Platelets play a vital role in blood clot formation and haemostasis, and their importance becomes especially evident in clinical situations such as trauma, cardiac surgery and haematological malignancies. Platelet transfusions are a key treatment for addressing low platelet counts, which can occur due to significant blood loss, dilutional coagulopathy or underlying disease processes and as a result, platelets are among the most frequently used blood products,^1^ with global demand steadily increasing due to advancements in surgical, oncological and critical care practices.^2^

Since the 1960s, platelets have been stored at room temperature (20–24°C) with agitation, limiting their shelf life to 5–7 days due to metabolic degradation and the risk of bacterial contamination. This short shelf life poses significant logistical challenges for blood suppliers and hospitals, leading to frequent shortages and wastage of up to 30% of platelet units due to expiration.^3^ The COVID-19 pandemic further highlighted these vulnerabilities, with acute platelet shortages reported in many regions.^4^ Cold-stored platelets (CSP), which were standard practice before the 1970s and are stored at 1–6°C, offer an alternative with extended shelf life (up to 21 days) and reduced bacterial contamination risks, though they have shorter post-transfusion survival compared with room-temperature platelets (RTP). CSP can either be apheresis derived or buffy coat derived.

The widespread adoption of RTP was driven by the need for longer post-transfusion survival in chronically thrombocytopenic patients, such as those undergoing chemotherapy. However, RTP have notable limitations, including bacterial contamination risks, suboptimal haemostatic function and unique storage conditions. While a significant proportion of platelet transfusions are used prophylactically in haematology-oncology patients, a substantial number are administered to actively bleeding patients, such as those with traumatic injuries, upper gastrointestinal bleeding or undergoing major surgery. These patients may benefit from CSP, which have been shown to possess enhanced haemostatic properties and superior clot-forming capabilities. Therefore, the expectation is that in bleeding patients there would be reduced mortality and/or morbidity following CSP transfusion. In addition, extended shelf-life and simplified storage conditions would enable platelet transfusion to be a viable therapy in more remote locations and thus a greater number of healthcare settings, improving healthcare equality.

In vitro evidence suggests that CSP may be particularly effective in the acute management of active bleeding, where rapid haemostasis is critical. Subsequently, several clinical trials investigating CSP have been conducted and recently reported.^5 6^ In addition, CSP have been used to improve supply chain resilience in some settings. Therefore, the timing is right to review this emerging evidence.

### Rationale

This review addresses a gap in understanding the therapeutic potential of CSP. While CSP offer promising advantages such as extended shelf life, reduced bacterial contamination and enhanced haemostatic properties, their clinical efficacy and safety remain underexplored compared with RTP.

### Aims

The aim of this systematic literature review and meta-analysis is to summarise the current evidence on the therapeutic use of CSP and to identify any reported safety concerns. By synthesising the available data, this review seeks to clarify the clinical role of CSP and provide insights to inform practice, guide future research and support the optimisation of transfusion strategies to improve patient outcomes.

## Methods and analysis

### Study design

This systematic review will explore the published literature on therapeutic transfusion of CSP. We will conduct a systematic review in accordance with the Cochrane Handbook for Systematic Reviews of Interventions^7^ methodological recommendations and will report as specified by the Preferred Reporting Items for Systematic Reviews and Meta-Analyses (PRISMA) statement.^8^ The protocol of the review is registered in PROSPERO (www.crd.york.ac.uk/prospero) CRD420251086610.

### Eligibility criteria

As we are interested in assessing all documented therapeutic use of CSP, our systematic review will include all published studies where CSP have been transfused in therapeutic doses, that is, where the intent is to treat or prevent bleeding (not given in microdoses to assess, eg, time in the circulation), including randomised controlled trials, feasibility studies, pilot trials and implementation reports/studies. CSP are defined as platelet concentrates stored at 1–6°C, that are derived from either apheresis or from buffy coat pooling. Studies examining delayed CSP, where the platelet concentrate is placed in the cold at >24 hours postmanufacture, will not be included in the review. The structured study question and inclusion and exclusion criteria are detailed in tables1  2.

**Table 1:** Population, Interventions, Comparisons, Outcomes (PICO) for study

Population	Patients requiring platelet transfusion as a therapeutic intervention
Intervention	CSP transfusion at treatment doses
Comparison	Transfusion of red cells/plasma with or without RTP
Outcomes	Primary outcomes—mortality measures Secondary outcomes—transfusion volume, adverse effects, functional outcome scores (eg, EQ5D5L, WHODAS2.0), intensive care days, length of hospital stay, laboratory testing and antibiotic use CSP storage duration and wastage Health economic analysis
Study design	Clinical trials (randomised and non-randomised), feasibility studies, pilot studies or implementation reports/studies. Studies will be included with direct comparator arms to RTP as well as single-arm CSP studies and implementation studies without a concurrent comparator

CSP, cold-stored platelets; EQ5D5L, EuroQoL 5-Dimension 5-Level; RTP, room-temperature platelets; WHODAS2.0, World Health Organization Assessment Schedule 2.0.

**Table 2:** Eligibility criteria

Inclusion criteria	Detail transfusion of CSP stored between 1°C and 6°C for therapeutic use in humans (clinical trials, feasibility studies or pilot studies)
	Contain human participants, with no age limit
	Full-text available
	Written in English language (no translation service available)
Exclusion criteria	Do not use therapeutic doses of CSP (ie, microdosing studies)
	Studies using healthy volunteers
	Studies using CSP within whole blood
	Delayed cold storage of platelets (>24 hours postmanufacture)

CSP, cold-stored platelets.

### Information sources

We will systematically search MEDLINE and Embase on the OVID platform, the Cochrane Library, Scopus, the Transfusion Evidence Library, the WHO International Clinical Trials Registry Search Portal (WHO, apps.who.int/trialsearch/) and the ClinicalTrials.gov website (US National Institutes of Health,www.clinicaltrials.gov/ from inception for eligible studies. We will also perform a grey literature search through any key organisational websites identified, in addition to searching the reference lists of all included studies for additional relevant studies.

### Search strategy

Our search strategies will use a combination of database-specific controlled vocabulary and keywords, including any relevant synonyms, relating to the transfusion of CSP. The search will initially be designed in Medline and translated for use in the other databases. No date restrictions will be applied, but studies will be limited to the English language only due to the lack of translation services. The MedlineALL (OVIDSP) search strategy is presented in online supplemental appendix 1. This strategy has been tested using preidentified pertinent papers to ensure relevant studies are being captured. Results will be imported into EndNote (V.21, Clarivate, Philadelphia, PA, 2013), and duplicates will be removed. References will then be exported into Rayyan^9^ for independent screening.

### Study selection

The search strategy will be developed and performed by a trained librarian. All identified abstracts will be independently screened by two reviewers to assess eligibility based on the criteria in table 2. Discrepancies in study selection will be resolved through discussion, with arbitration by a third reviewer when consensus cannot be reached. For studies meeting the eligibility criteria at the abstract screening stage, full-text articles will be independently assessed by the same two reviewers against the same eligibility criteria, with a third reviewer consulted as an adjudicator when necessary.

Once we have the definitive papers for the review, we will begin the data extraction process.

### Data extraction

Data will be extracted into an Excel data sheet template by two independent reviewers to confirm that the data extracted is correct. Differences in data will be adjudicated by a third reviewer. Data items to be extracted are summarised in table 3.

**Table 3:** Data to be extracted from eligible studies

Domain	Key items
Study design	Source of data (eg, cohort, case–control, clinical trial (randomised and non-randomised), pilot study)
	Blinding procedures (single blind, double blind, open-label)
	Randomisation type (simple, block, cluster), stratification and/or minimisation factors, allocation concealment, timing
	Location and setting of study, and number of sites
Participants	Participant eligibility and recruitment method (eg, inclusion and exclusion criteria)
	Participant description (eg, demographics, patient history, physical examination, additional testing, disease characteristics)
	Method of consent (eg, presumed, family, patient)
	Study dates and/or recruitment timeframe
	Bleeding indication (eg, trauma, gastrointestinal, other)
Intervention/comparator	Details of treatments received: timing of intervention, whether room-temperature platelets were also transfused and the indication for transfusion
	Details about cold-stored platelets manufacture: method of manufacture, suspension medium, shelf life, storage temperature
Outcome(s) measured	Was the same outcome definition (and method for measurement) used in all patients?
	Primary and secondary outcomes
	Type of outcome (eg, single or combined endpoints)
	Was the outcome assessed without knowledge of the candidate predictors (ie, blinded)?
	Time of outcome occurrence or summary of duration of follow-up
Sample size	Number of participants and number of outcomes/events
	Calculation method (eg, power level, significance level)
	Outcomes/events and effect size used for estimation
	Attrition rates
Missing data	Number of participants with any missing value (include predictors and outcomes)
	Number of participants with missing data for each predictor
	Handling of missing data (eg, complete-case analysis, imputation or other methods)
Analysis methods/evaluation	Types of models used for analysis
	Analysis population and analysis type (eg, intention-to-treat, per protocol, complete case)
	Identify numerical results (eg, mortality rates, risk ratios, OR)
	Level of statistical significance (eg, p values, CI ranges)
	Safety (eg, (serious) adverse events)
	Subgroup findings (eg, age, sex, injury type and/or severity)
Interpretation and discussion	Summary of presented results
	Interpretation of presented results (confirmatory, ie, effectiveness and safety of intervention demonstrated—more research needed)
	Comparison with other studies, discussion of generalisability, strengths and limitations

### Risk of bias

Two authors will independently assess the risk of bias for the included studies. In cases of disagreement, items will be discussed with an adjudicator. Risk of bias will be assessed using the risk-of-bias tool for randomised trials (RoB-2), recommended for assessment of bias in randomised control trials included in Cochrane reviews. Where non-randomised studies are included in the review, we will consider appropriate bias assessment tools (eg, the Risk of Bias In Non-randomized Studies - of Interventions).^10^

### Data synthesis and analysis

Studies will be grouped and synthesised according to key characteristics, including clinical indication. Randomised and non-randomised studies will be analysed separately to account for differences in study design and risk of bias. Statistical heterogeneity will be assessed using the I² statistic. Where outcome data are sufficiently comparable across studies (I² ≤50%), a fixed-effects model meta-analysis will be undertaken. For I² between 50% and 75%, a random-effects model will be used. Where heterogeneity is considerable (I² >75%), the appropriateness of meta-analyses will be assessed. A narrative synthesis will be undertaken should heterogeneity across studies be deemed too substantial to permit meaningful meta-analysis (I^2^ ≥75%), or where significant concerns regarding risk of bias are identified. Box and whisker plots will be used to demonstrate the distribution and variability across studies.

The primary outcome of interest is mortality at defined timepoints (eg, 6 hours, 24 hours and 30 days) in patients receiving CSP, with further consideration of relevant secondary outcomes (eg, length of hospital stay and transfusion volume). Mortality outcomes reported at different timepoints will be analysed separately.

Effect estimates for dichotomous outcomes (including mortality and adverse events) will be summarised using risk ratios (RRs), ORs or HRs with 95% CIs. Continuous outcomes will be summarised using mean differences or non-parametric alternatives. Studies with zero events in one arm will be included using a continuity correction to retain data and statistical power, whereas studies with zero events in both arms will be described narratively.

The primary outcome of interest is mortality at various timepoints (eg, 6 hours, 24 hours and 30 days) in patients receiving CSP, with further consideration of other relevant secondary outcomes. Effect estimates for dichotomous outcomes will be presented as RRs, ORs or HRs with 95% CIs. Forest plots will be used to visualise pooled results.

Where sufficient data are available, prespecified subgroup analyses will explore differences in treatment effects of CSP by clinical indication including traumatic bleeding, gastrointestinal bleeding and surgical bleeding. Stratified meta-analyses will be used where appropriate; otherwise, narrative syntheses will be conducted.

The Grading of Recommendations Assessment, Development and Evaluation (GRADE)^11^ approach will be used to evaluate the certainty of evidence across outcomes. As above, multiple independent reviewers will assess GRADE outcomes.

## Ethics and dissemination

No ethical review is required as no original data will be collected. We aim to publish the results of this review in an appropriate peer-reviewed journal. We will also submit to present the findings at an appropriate conference.

## Supplementary material

10.1136/bmjopen-2025-113548 online supplemental file 1

## References

[1] Lu J, Karkouti K, Peer M (2023). Cold-stored platelets for acute bleeding in cardiac surgical patients: a narrative review. Can J Anesth/J Can Anesth.

[2] Estcourt LJ (2014). Why has demand for platelet components increased? A review. Transfus Med.

[3] (2020). International Society of Blood Transfusion, The 36th International ISBT Congress, virtual meeting, 12-16 December 2020. Vox Sang.

[4] Warner MA, Kurian EB, Hammel SA (2021). Transition from room temperature to cold-stored platelets for the preservation of blood inventories during the COVID-19 pandemic. Transfusion.

[5] Sperry JL, Guyette FX, Rosario-Rivera BL (2024). Early Cold Stored Platelet Transfusion Following Severe Injury: A Randomized Clinical Trial. Ann Surg.

[6] Neal MD, Okonkwo DO, Guyette FX (2025). Early Cold-stored Platelet Transfusion following Traumatic Brain Injury: A Randomized Clinical Trial. Ann Surg.

[7] Higgins JPT, Chandler J, Cumpston M (2024). Cochrane Handbook for Systematic Reviews of Interventions.

[8] Page MJ, McKenzie JE, Bossuyt PM (2021). The PRISMA 2020 statement: an updated guideline for reporting systematic reviews. BMJ.

[9] Ouzzani M, Hammady H, Fedorowicz Z (2016). Rayyan-a web and mobile app for systematic reviews. Syst Rev.

[10] Sterne JA, Hernán MA, Reeves BC (2016). ROBINS-I: a tool for assessing risk of bias in non-randomised studies of interventions. BMJ.

[11] Balshem H, Helfand M, Schünemann HJ (2011). GRADE guidelines: 3. Rating the quality of evidence. J Clin Epidemiol.

