# Supplementary material for: Therapeutic use of cold-stored platelets in humans: protocol for a systematic review and meta-analysis
Source: BMJ Open. 2026 Jun 29;16(6):e113548. doi: 10.1136/bmjopen-2025-113548 (PMC13343117; doi:10.1136/bmjopen-2025-113548)
Supplement: online supplemental file 1 [file bmjopen-16-6-s001.docx]

**Appendix I: Search Strategy for MEDLINE**

1. Blood Platelets/

2. (platelet* or thrombocyte*).tw,kf.

3. 1 or 2

4. Cold Temperature/

5. Refrigeration/

6. (Cold store* or cold storage).tw,kf.

7. 4 or 5 or 6

8. 3 and 7

9. ((cold or refrigerat*) adj4 (platelet* or thrombocyte*)).tw,kf.

10. 8 or 9

11. exp Platelet Transfusion/

12. (platelet* adj4 (transfus* or treatment*)).tw,kf.

13. (transfus* adj3 (bleed* or haemorrhag* or hemorrhag* or trauma or prophylaxis or surger* or injur*)).tw,kf.

14. or/11-13

15. 10 and 14

16. exp animals/ not humans.sh.

17. 15 not 16

18. limit 17 to english language
