# Supplementary material for: Therapeutic use of cold-stored platelets in humans: protocol for a systematic review and meta-analysis
Source: BMJ Open. 2026 Jun 29;16(6):e113548. doi: 10.1136/bmjopen-2025-113548 (PMC13343117; doi:10.1136/bmjopen-2025-113548)
Supplement: Supplementary file 2 [file bmjopen-2025-113548.reviewer_comments.pdf]

PEER REVIEW HISTORY

BMJ Open publishes all reviews undertaken for accepted manuscripts. Reviewers are asked to complete a checklist review form and are provided with free text boxes to elaborate on their assessment. These free text comments are reproduced below.

ARTICLE DETAILS

Title (Provisional)

Therapeutic use of cold stored platelets in humans: protocol for a systematic review and meta-analysis

Authors

George, Chloë E; Scorer, Thomas; Cleves, Anne; Pilavakis, Ioannis; Harris, Shaun; Carter, Kymberley

---

VERSION 1 - REVIEW

**Reviewer**	**1**
**Name**	**Stone, Elizabeth**
**Affiliation**	**Columbia University, Department of Pathology & Cell Biology**
**Date**	**26-Feb-2026**
**COI**	**None**

C. George and colleagues have submitted a nice protocol report detailing their plans for a systemic review and meta-analysis on the use of cold stored platelets (CSP). This is a much-needed study to attempt to aggregate what has been perviously published regarding CSP to help define whether there are situations where CSP may have a therapeutic use. The authors note the limitation that there are few RCTs published regarding CSP, and they may have to perform a narrative synthesis of the available studies instead, which is my one concern for this review. Otherwise, this manuscript is very well written and well organized, and their protocol is well thought out. I look forward to the results of this study. I only have one comment:

• Table 3, page 6, lines 40-42: the first Key Item under Intervention/Comparator appears incomplete with the text cut off. Please address this in the resubmission.

**Reviewer**	**2**
**Name**	**Chen , pu**
**Affiliation**	**Zhongshan Hospital**

Date **24-Mar-2026**

COI **None**

This work focuses on the therapeutic use of cold stored platelets (CSP), an urgent need in transfusion medicine that could optimize platelet supply chains and improve haemostatic care for bleeding patients. As the first systematic review/meta-analysis on therapeutic CSP, it fills a key evidence gap, adheres to Cochrane/PRISMA-P guidelines with PROSPERO registration, uses rigorous bias assessment (RoB-2/ROBINS-I) and GRADE methods, and selects clinically meaningful outcomes to guide transfusion practice.

Limitations:

1.To provide guidance for clinical practice, the specific temperature range for CSP preservation must be explicitly defined. A statement should be added in the Limitations section to acknowledge the regrettable absence of a subgroup comparison of CSP-related data across different storage temperature ranges.

2.No pre-specified stratification analysis has been planned for specific clinical populations (e.g., patients with traumatic bleeding, gastrointestinal bleeding, or those undergoing cardiac surgery). Given that the therapeutic efficacy of CSP may differ significantly in patients with distinct bleeding aetiologies.

3.The manuscript does not specify a clear numerical threshold for the I^2 statistic (e.g., whether a random-effects model or narrative synthesis will be applied when I^2 exceeds 50% or 75%), which leads to inadequate practical operability for the execution of the planned statistical analysis.

Reviewer **3**

Name **Kuwatsuka, Yachiyo**

Affiliation **Nagoya University Hospital, Center for Advanced Medicine and Clinical Research**

Date **25-Mar-2026**

COI **None**

This manuscript is a protocol for a systematic review and meta-analysis on the therapeutic use of cold-stored platelets, with PROSPERO registration, study selection and data extraction conducted independently by two reviewers, and the planned use of established methodological tools. This is a clinically relevant topic in transfusion medicine, and the manuscript has several strengths. However, several methodological details require clarification.

My main comments are as follows:

1) The protocol states that it will assess the therapeutic use of cold-stored platelets in humans broadly, but Table 1 lists room-temperature platelets as the comparator, while the data synthesis section identifies mortality at various timepoints in trauma patients as the primary outcome. Please clarify whether the review is intended to cover all therapeutic indications or primarily trauma, and whether only studies with a direct room-temperature platelet comparator will be eligible, or whether single-arm cold-stored platelet studies and implementation studies without a concurrent comparator will also be included.

2) It would be helpful to prespecify what qualifies as a cold-stored platelet product, including storage temperature range and other relevant manufacturing characteristics. Please also define what is meant by “therapeutic doses”.

3) Considerable clinical and methodological heterogeneity is likely across indications, study designs, and platelet-processing methods. Please clarify how studies will be grouped and synthesized, for example by clinical indication. In particular, please state whether randomized and non-randomized studies will be analyzed separately and under what circumstances pooling would be considered appropriate.

4) It would be helpful to further clarify the planned statistical analysis, including the preferred summary measure for each outcome, the approach to multiple mortality timepoints, the handling of zero-event studies, and any planned sensitivity analyses. The criteria for choosing between fixed-effect and random-effects models should be more clearly described.

Minor comments

Please standardize minor style issues, for example “RCTs” rather than “RCT’s,” throughout the manuscript.

VERSION 1 - AUTHOR RESPONSE

Reviewer: 1
Dr. Elizabeth Stone, Columbia University
Comments to the Author:
C. George and colleagues have submitted a nice protocol report detailing their plans for a systemic review and meta-analysis on the use of cold stored platelets (CSP). This is a much-needed study to attempt to aggregate what has been perviously published regarding CSP to help define whether there are situations where CSP may have a therapeutic use. The authors note the limitation that there are few RCTs published regarding CSP, and they may have to perform a narrative synthesis of the available studies instead, which is my one concern for this review. Otherwise, this manuscript is very well written and well organized, and their protocol is well thought out. I look forward to the results of this study. I only have one comment:

• Table 3, page 6, lines 40-42: the first Key Item under Intervention/Comparator appears incomplete with the text cut off. Please address this in the resubmission.

Thank you for your comment, we have amended the text to ensure it reads as a complete sentence.

Reviewer: 2
Dr. pu Chen , Zhongshan Hospital
Comments to the Author:
This work focuses on the therapeutic use of cold stored platelets (CSP), an urgent need in transfusion medicine that could optimize platelet supply chains and improve haemostatic care for bleeding patients. As the first systematic review/meta-analysis on therapeutic CSP, it fills a key evidence gap, adheres to Cochrane/PRISMA-P guidelines with PROSPERO registration, uses rigorous bias assessment (RoB-2/ROBINS-I) and GRADE methods, and selects clinically meaningful outcomes to guide transfusion practice.
Limitations:
1.To provide guidance for clinical practice, the specific temperature range for CSP preservation must be explicitly defined. A statement should be added in the Limitations section to acknowledge the regrettable absence of a subgroup comparison of CSP-related data across different storage temperature ranges.

The specific temperature range has been added to the background information. An additional point has been added to the strengths and limitation of the study to detail that no subgroup analysis of CSP related data across different storage temperature ranges will be performed.

2.No pre-specified stratification analysis has been planned for specific clinical populations (e.g., patients with traumatic bleeding, gastrointestinal bleeding, or those undergoing cardiac surgery). Given that the therapeutic efficacy of CSP may differ significantly in patients with distinct bleeding aetiologies.

We agree that the efficacy and safety profile of CSP may differ across patients with distinct bleeding aetiologies. We have revised the protocol to incorporate pre-specified sub-group analyses. We are aware that the evidence base for some sub-groups may be limited, therefore, studies will be stratified by major bleeding categories providing sufficient data are available. This will include traumatic bleeding, gastrointestinal bleeding, and surgical bleeding.

The subgroup analyses will explore heterogeneity and assess whether treatment effects differ across bleeding aetiologies.

3.The manuscript does not specify a clear numerical threshold for the I^2 statistic (e.g., whether a random-effects model or narrative synthesis will be applied when I^2 exceeds 50% or 75%), which leads to inadequate practical operability for the execution of the planned statistical analysis.

Thank you for highlighting the need for greater clarity regarding the I^2 statistic. We have revised the protocol to include pre-specified thresholds for interpretation and choice of analytical models. Where moderate to substantial heterogeneity is detected, we will preferentially use a random-effects model and further explore sources of heterogeneity through subgroup analyses. Should heterogeneity not be adequately explained, meta-analyses will not be conducted and we will use a narrative synthesis.

Reviewer: 3
Dr. Yachiyo Kuwatsuka, Nagoya University Hospital
Comments to the Author:
This manuscript is a protocol for a systematic review and meta-analysis on the therapeutic use of cold-stored platelets, with PROSPERO registration, study selection and data extraction conducted independently by two reviewers, and the planned use of established methodological tools. This is a clinically relevant topic in transfusion medicine, and the manuscript has several strengths. However, several methodological details require clarification.

My main comments are as follows:
1) The protocol states that it will assess the therapeutic use of cold-stored platelets in humans broadly, but Table 1 lists room-temperature platelets as the comparator, while the data synthesis section identifies mortality at various timepoints in trauma patients as the primary outcome. Please clarify whether the review is intended to cover all therapeutic indications or primarily trauma, and whether only studies with a direct room-temperature platelet comparator will be eligible, or whether single-arm cold-stored platelet studies and implementation studies without a concurrent comparator will also be included.

Table 1 updated to amend the comparator to transfusion of red cells/plasma with or without RTP. Study design in PICO also updated to include studies which have both a direct comparator (RTP) and single-arm CSP studies/implementation studies without a comparator arm. The review is intended to cover all therapeutic indications for CSP not only trauma, this has been amended in the 'Data synthesis and analysis' section.

2) It would be helpful to prespecify what qualifies as a cold-stored platelet product, including storage temperature range and other relevant manufacturing characteristics. Please also define what is meant by “therapeutic doses”.

Storage temp of CSP (1-6°C) added to Table 2 and into background section. Manufacturing methods also added to background and eligibility criteria: CSP can be apheresis or buffy coat derived and delayed cold stored platelets , where the platelet concentrate is placed in the cold at >24 hours post-manufacture will not be included in the review. Definition of therapeutic dose added to the eligibility criteria.

3) Considerable clinical and methodological heterogeneity is likely across indications, study designs, and platelet-processing methods. Please clarify how studies will be grouped and synthesized, for example by clinical indication. In particular, please state whether randomized and non-randomized studies will be analyzed separately and under what circumstances pooling would be considered appropriate.

We have highlighted our approach in dealing with heterogeneity in our response to reviewer 2 comment 3. We have amended the protocol to clarify that studies will be stratified by key clinical indication. Additionally, randomised and non-randomised studies would be analysed separately. Pooling of results will only be undertaken if the level of heterogeneity is low to moderate ($I^2 \leq 50\%$).

4) It would be helpful to further clarify the planned statistical analysis, including the preferred summary measure for each outcome, the approach to multiple mortality timepoints, the handling of zero-event studies, and any planned sensitivity analyses. The criteria for choosing

between fixed-effect and random-effects models should be more clearly described.

We have expanded the statistical analysis section to ensure that the planned statistical analyses are clearer including the handling of multiple mortality timepoints and zero-event studies.

We have added clearer criteria for the choice of fixed-effect and random-effect models based on the degree of heterogeneity in response to reviewer 2 comment 3.

Minor comments

Please standardize minor style issues, for example “RCTs” rather than “RCT’s,” throughout the manuscript.

This has been addressed.
